# Enhancing Pollinator Support: Plant–Pollinator Dynamics Between *Salvia yangii* and *Anthidium* Bees in Anthropogenic Landscapes

**DOI:** 10.3390/biology14081084

**Published:** 2025-08-19

**Authors:** Daniela Lupi, Claudia Giuliani, Gelsomina Fico, Serena Malabusini, Carla Sorvillo, Manuela Giovanetti

**Affiliations:** 1 Department of Food Environmental and Nutritional Sciences, University of Milan, 20133 Milan, Italy; serena.malabusini@unimi.it (S.M.); carla.sorvillo@unina.it (C.S.); 2 Department of Pharmaceutical Sciences, University of Milan, 20133 Milan, Italy; claudia.giuliani@unimi.it (C.G.); gelsomina.fico@unimi.it (G.F.); 3 Ghirardi Botanical Garden, Department of Pharmaceutical Sciences, University of Milan, Via Religione 25, Toscolano Maderno, 25088 Brescia, Italy; 4 Department of Biology, University of Federico II of Naples, 80126 Naples, Italy; 5 NBFC—National Biodiversity Future Center, 90133 Palermo, Italy; 6 CREA Research Centre for Agriculture and Environment, 40128 Bologna, Italy; manuela.giovanetti@gmail.com

**Keywords:** urban-rural environments, ornamental flower resources, trichomes, flower visits, wild bees, honey bees

## Abstract

Anthropogenic environments are increasingly recognised for their potential to support pollinator diversity, especially through the strategic selection of ornamental plant species. This study investigated the ecological role of *Salvia yangii* (formerly *Perovskia atriplicifolia*) in supporting solitary bees, particularly species of the genus *Anthidium*, within urban green spaces in Milan (Italy). Field observations were conducted in both urban and rural sites to assess pollinator visitation rates, bee abundance, and plant traits relevant to nesting and foraging. *S. yangii* attracted a diverse assemblage of bee genera, with *Anthidium* spp. consistently recorded as dominant visitors. This preference is likely driven by the short corolla tube, which facilitates the access to nectar, and its dense coverage of trichomes, which may serve as essential nesting materials. The interaction was found to be stable across environments, suggesting that *S. yangii* provides reliable floral and structural resources. These findings highlight the potential of specific ornamental plants to contribute meaningfully to urban pollinator conservation. While the use of exotic species warrants caution, we propose that *S. yangii* may be suitable for inclusion in pollinator-friendly planting guidelines under controlled horticultural conditions, offering a promising model for enhancing urban biodiversity through informed plant selection.

## Simple Summary

As cities grow, finding ways to support bees and other pollinators becomes more important for healthy ecosystems and food production. The present study focused on the role of the Russian sage (*Salvia yangii*), a popular garden plant, in supporting bees in Milan, Italy. The plant attracted many bees, and *Anthidium* species were the most frequent visitors. These bees are attracted not only to the nectar, which is easy to reach thanks to the flower’s shape, but also to the tiny hairs on the plant that *Anthidium* spp. use to build their nests. This beneficial relationship between the plant and bees was observed in city parks and rural areas. The reliable floral and structural resources of *S. yangii* make it valuable for supporting local bees in urban gardens and green spaces. Although care must be taken when introducing exotic plants, this species shows promise as an environmentally friendly gardening choice that benefits both people and wildlife.

## 1. Introduction

Anthropogenic environments are increasingly recognised as important habitats for a variety of solitary bee species, many of which show high adaptability to fragmented and human-modified landscapes. Among the most commonly observed are species of the genera *Osmia* Panzer, 1806 (mason bees), *Megachile* Latreille, 1802 (leafcutter bees), and *Anthidium* Fabricius, 1804 (wool-carder bees), all of which are members of the family Megachilidae [1,2]. These bees frequently nest in cavities provided by wood, plant stems, or artificial substrates such as bee hotels, and are often associated with both native and ornamental plant species commonly found in gardens and urban parks. Their generalist foraging behaviour and nesting plasticity make them particularly successful in cities, where they can exploit a wide range of floral and nesting opportunities [3].

The bee genus *Anthidium* (tribe Anthidiini) comprises approximately 160 recognised species, which are distributed across all continents and are relatively evenly represented in both the Eastern and Western Hemispheres [4,5,6]. The widespread distribution of the genus is likely facilitated by its flexible nesting requirements: nests are typically situated in cavities elevated above ground level and are lined with trichomes from woolly plants, such as species of *Salvia* L. [4,7]. *Anthidium* species are generalists in their foraging behaviour, attracted to a broad variety of flowering plants. However, preferred floral resources are rarely specified, with most reports indicating frequent visits to plants in the Fabaceae and Lamiaceae families. Graham et al. [8] suggested that *Stachys byzantina* K. Koch (commonly known as lamb’s ear) may attract *Anthidium* bees due to the structure of its leaf trichomes and potentially their secretions. Urban and suburban environments are often preferred reproductive habitats. They are commonly observed in urban parks, residential gardens, and even on green rooftops [9,10,11]. Due to their behavioural plasticity, *Anthidium* species serve as excellent models for investigating the role and importance of plant species in shaping pollinator communities.

Many *Salvia* species are commonly used as ornamentals in urban green spaces, providing a valuable nectar and pollen source for a wide range of pollinators [12,13]. *Salvia* species are popular for their vivid flowers, attractive foliage, and adaptability across various garden settings. Their blooms, in hues ranging from blues and purples to reds and whites, create visually striking displays that attract a range of pollinators, including bees, butterflies, and hummingbirds. Frequently recommended in gardening manuals [14], salvias are employed in borders, beds, container gardens, and as ground covers. Some species are also favoured in xeriscaping due to their drought tolerance. Their low maintenance requirements and adaptability to diverse climates make them a practical and aesthetically appealing choice for gardeners. A notable example is *Salvia yangii* B. T. Drew (formerly *Perovskia atriplicifolia* Benth,. Lamiaceae), known as Russian sage, which is native to rocky regions in Pakistan, Afghanistan, Iran, and Tibet. It has been widely adopted as an exotic ornamental in urban landscapes and private gardens worldwide.

Plant trichomes are hypothesised to play multiple, and at times contradictory, roles in plant–insect interactions. On the one hand, trichomes may facilitate insect attachment to plant surfaces, with some insect species having evolved specialised claws to grip them [15]. On the other hand, trichomes can function as direct or indirect defences against herbivores [16]. Trichomes of *Salvia* species have been extensively studied in this context, particularly with respect to their morphology and secretory functions, and their relationship with visiting insects [17,18]. In addition, *Salvia* species have been investigated for their pollination ecology in relation to floral morphology ([19] and references therein).

As part of a broader project aimed at identifying anthropogenic habitats in Milan (Italy) that support Apoidea and informing guidelines for pollinator-friendly planting, this study specifically aimed to investigate the plant–pollinator dynamics between *Anthidium* spp. and the widely planted ornamental species *S. yangii*. Baldock et al. [2] found *Anthidium* among the core group of urban pollinators in UK cities, showing their relevance in maintaining urban pollination services. Similarly, Geslin et al. [20] documented *Anthidium* spp. interacting with a variety of ornamental and native plants in Mediterranean urban parks, indicating their adaptability to human-modified habitats. In North America, Russo et al. [21] observed *Anthidium manicatum* L. within highly nested plant–pollinator networks in community gardens, noting its frequent interactions with both native and exotic floral species. These studies highlight *Anthidium*’s flexibility and contribution to the plant–pollinator network, pointing to a possible central role of this genus. A comprehensive survey of urban green spaces was carried out to record *Anthidium* spp. visitation, assess abundance, and examine the plant’s trichome morphology and secretory activity. These data were integrated with some records of other flower visitors sharing the same niche. To test the robustness of the *Anthidium–Salvia* interactions in anthropogenic contexts, observations were also replicated in a rural setting, allowing for the assessment of the suitability of this system as a model for pollination ecology.

## 2. Materials and Methods

### 2.1. Study Sites

#### 2.1.1. The Urban Area

**Figure 1:**
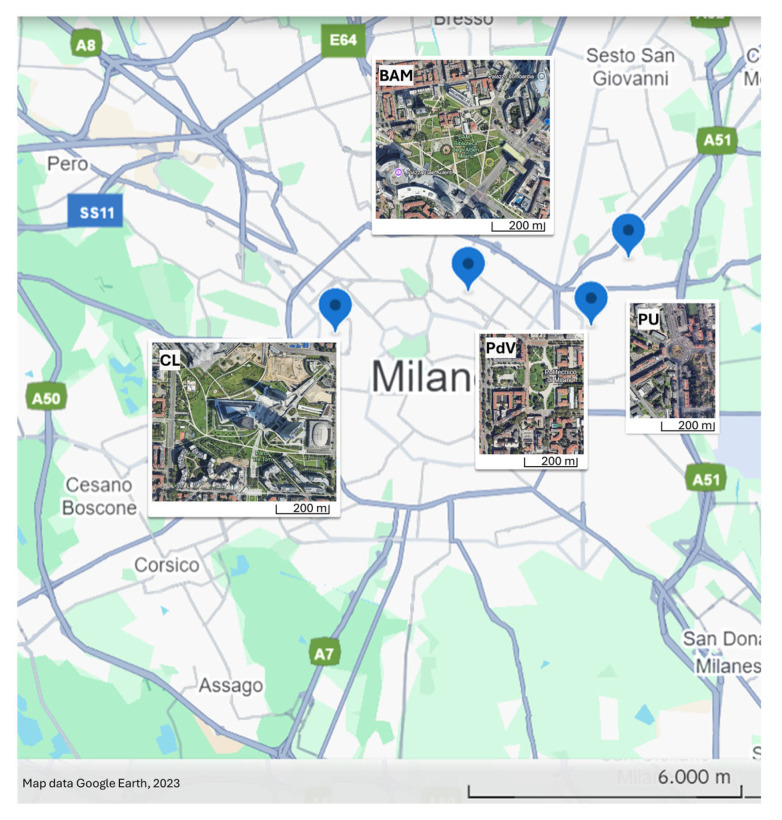
Street map of Milan, with position and satellite views of the four green areas. Abbreviations refer to the following sites: Piazza Leonardo da Vinci (PdV), Parco City Life (CL), Biblioteca degli Alberi (BAM), and Piazzale Udine (PU).

According to analyses conducted by the Municipality of Milan (available at https://geoportale.comune.milano.it/sit/, accessed on 17 July 2025), the urban landscape is characterised by fragmented green areas, although, from 2011 to 2016, there was a consistent annual increase of 2–3% in green space. In addition to formal green spaces, the city also contains a network of tree-lined streets, avenues, and roundabouts. While these are not officially classified as greening infrastructure (e.g., parks or communal gardens), they are ecologically relevant to flying insects. These areas often feature herbaceous alien ornamental species, with *S. yangii* being among the most commonly planted in recent years. The study involved 4 sites (Figure 1): Parco City Life (CL) 45°28′38″ N 9°09′22″ E, Piazza Leonardo da Vinci (PdV) 45°28′40″ N 9°13′34″ E, Biblioteca degli Alberi (BAM) 45°29′02″ N 9°11′32″ E, and Piazzale Udine (PU) 45°29′27″ N 9°14′12″ E (Figure 1). There was at least a 1 km distance from one site to the other; at all sites, *S. yangii* was included among other ornamentals.

#### 2.1.2. The Rural Area

An extra site in a rural area was added (45°17′25.4″ N 11°30′09″ E), where *S. yangii* plants were positioned in a private garden. The rural area was quite distant from Milan (about 200 km) and *S. yangii* was not used as ornamental in the nearby areas. Plants were positioned in May 2021 and left in place during a flowering season.

### 2.2. The Plant Species

*Salvia yangii* is a deciduous perennial subshrub, which typically grows to a height of 0.5–1.2 m. The stems are erect, woody at the basal portion and square in cross-section at the terminal portion, with a silvery appearance. The greyish-green and aromatic leaves are shortly petiolate and are arranged in opposite pairs; the overall shape is oblate to lanceolate with a deeply incised margin that may be either wavy or sharp-toothed. The flowers are arranged into showy, branched inflorescences, 30–38 cm long; the calyx is purple, densely covered in hairs; the hairy corolla, blue to violet in colour, is tube-shaped, formed from a four-lobed upper lip and a slightly shorter lower lip; the blooming period lasts all summer.

#### 2.2.1. Visual Display and Flower Morphology

At the urban area, the plant was commonly distributed in patches or linear corridors, frequently outcompeting other floral resources or occurring as the only available floral source. At each patch, we estimated the visual display: in a sub-area of 50 cm × 50 cm, we counted all flowering stems, and out of five of them (randomly selected), we recorded the number of flowers on each stem. Flowers were also investigated in their morphology. Thirty randomly selected fully-opened flowers per plant, collected at the urban area, were dissected and measured using a digital calliper and a stereomicroscope. Five floral morphological traits were selected and measured the following: (i) calyx length; (ii) flower length; (iii) upper lip length; (iv) lower lip length; (v) length of the corolla tube (measured as the distance between the top of the ovary—where the nectary is typically located—and where the petals separate).

At the rural area, some records were replicated on plants added to the garden: the visual display in a sub-area of 50 cm × 50 cm, counting stems, and recording the number of flowers on each stem.

#### 2.2.2. Trichome Analyses

The structure, the distribution pattern, and the histochemistry of the secretory structures on the vegetative and reproductive organs were described by means of scanning electron microscopy (SEM), light microscopy (LM), and fluorescence microscopy (FM). For each examined plant part, at least ten replicates from the urban area were examined to evaluate the level of variability of the micromorphological features.

For SEM we collected leaves, calyces, and corollas, hand-prepared and fixed in FAA solution (formaldehyde/acetic acid/ethanol 70% = 5:5:90) for 24 h, dehydrated in an ascending ethanol series up to absolute, and critical-point dried. The samples were mounted on aluminium stubs and gold-coated. Observations were performed under a Zeiss^®^ EVO MA15 SEM (Zeiss, Oberkochen, Germany) operating at 10 kV at the Interdepartmental Center for Electron Microscopy and Microanalysis Services (M.E.M.A.) of the University of Florence (Florence, Italy).

For LM and FM, we employed the same above-mentioned plant parts, conducting a micromorphological survey. We used both fresh material and fixed samples included in historesin (Technovit^®^ 7100, Heraeus Kulzer GmbH & Co. KG, Wehrheim, Germany). For details on fresh plant material, see Appendix A.

### 2.3. Bee Records

#### 2.3.1. Bees at the Urban Area

Trained observers carried out patch records (n = 388) during three years (2019, 2020, and 2021) between May and September, for a total of 1940 min (more than 32 h) and 5819 records of Apoidea. During ten patch records, no bees were observed visiting the flowers. The observed patch area was 1 m × 1 m: a patch was observed once for a 5 min period, and patches changed during following observations; time of the day varied from 8:00 to 19:00. All bee pollinators visiting the patch area were recorded after visual identification to the genus level upon contact with the flowers. If identification was positive, we did not catch the individual but visually followed its activity. When identification in the field was not possible, individuals were captured using vials for closer examination. If identification still could not be completed in the field, the specimens were brought to the laboratory for subsequent identification. As the observations were conducted over a short period of time and in a limited area, it was easy for the observer to detect repeated visits by the same individual, thus avoiding duplicate counts of the same bee. Temperature during observations ranged from 15.3 °C to 35 °C.

In addition to estimating the foraging behaviours of visiting bee species, at the urban area, focal observations were conducted on randomly selected individuals of *Anthidium* spp. (both males and females). During each observation, a single bee was visually classified, sexed, and tracked, and its activity was recorded for a duration of one minute. These observations enabled the documentation of bee movement across inflorescences, the number of flowers and inflorescences visited, and the type of resource collected (pollen or nectar). A total of 101 individuals, comprising 44 females and 57 males, were observed.

#### 2.3.2. Bees at the Rural Area

At the rural area, records were replicated by carrying out random patch records (n = 16) on pollinator visits in June, July, and August 2021, for a total of 80 min and 108 records of Apoidea. Temperature during observations ranged from 26.0 °C to 34.8 °C.

### 2.4. Statistical Analyses

The data on flower morphology were analysed using the analysis of variance (ANOVA), after the 30 replicates for each parameter had been transformed using the arcsine square root transformation (arcsin $\sqrt{x}$) for normalisation. Mean values and confidence intervals (α = 0.05) were obtained. The averages were then separated using Tukey’s B post hoc test; *p* < 0.05 was used to determine the significance of the differences between the means. Statistical analyses were performed using the JMP software package (v14.0.0, SAS Institute, Cary, NC, USA).

Data from patch records on pollinators were analysed using ANOVA and Tukey’s post hoc test; when the assumptions for ANOVA were not met, a non-parametric ANOVA and Kruskal–Wallis test were applied. The relative abundance (RA%) of the different bee genera was calculated following Layek et al. [22]. For the evaluation of the percentage data, angular transformation was used. To evaluate the effect of the estimated number of flowers in each observation patch on the number of different genera pollinators, Spearman’s rank correlations *stats* R package was used (Rstudio v4.4.) [23]. Additionally, partner diversity as the mean Shannon’s diversity index for interactions was calculated in each area [24].

## 3. Results

### 3.1. The Plant Species

#### 3.1.1. Visual Display and Flower Morphology

The average number of flowering stems exhibited considerable variability across months, years, and study areas. This outcome was anticipated, as numerous factors, such as irrigation, light exposure, soil nutrient availability, and temperature, can influence flowering dynamics. Overall, the plant showed a general increase in the average number of flowering stems, typically peaking in July, followed by a rapid decline. An exception to this pattern occurred in 2021, when July did not represent the peak flowering period. That year was characterised by a general reduction in floral abundance across all months. The estimated number of flowering stems ranged from 0 (May–June 2020) to over 3500 (July 2020) within a 1 m^2^ area.

Flower morphology is further described by the data in Table 1.

#### 3.1.2. Trichome Analyses

**Figure 2:**
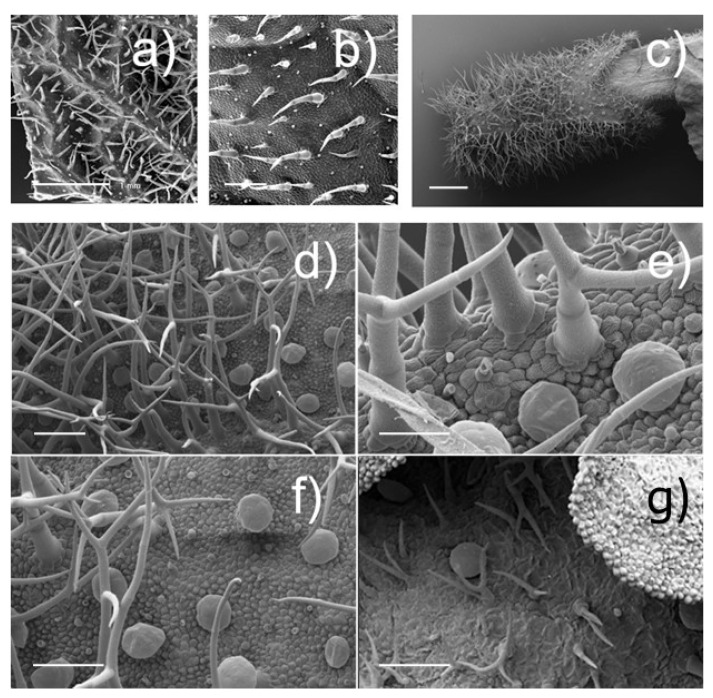
Non-glandular and glandular *indumenta* observed on *Salvia yangii* leaves, calyces, and corollas. (**a**) Lower leaf part and (**b**) upper leaf part; (**c**,**d**) calyx, abaxial side: general view (**c**) and details (**d**), with dendritic non-glandular hairs, peltates, and short-stalked capitates; (**e**–**g**) corolla, abaxial side: details of the abaxial side of the lower lip (**e**), of the tube (**f**), and of the upper lip (**g**) with dendritic non-glandular hairs, peltates, and short-stalked capitates. [*Scale bars: 1 mm (**a**); 200 μm (**b**); 500 μm (**c**); 100 μm* (***d**,**f**,**g***); *50 μm (**e**).*].

Leaves, calyces, and corollas of *S. yangii* were densely covered by non-glandular and glandular hairs (Figure 2). Non-glandular ones, present on all of the examined plant parts, with the exception of the calyx adaxial surface, were multicellular and dendritic or star-shaped: they were formed by a primary axis from which several secondary branches arose; each arm was uniseriate with a pointed apex. Two morphotypes of glandular trichomes were distinguished: peltate and short-stalked capitate. The peltate ones were composed by a basal cell, a neck cell, and a broad multicellular secretory head (40–50 μm in diameter). They occurred only on leaf abaxial lamina prevailing on the interveinal regions and on the abaxial surfaces of the calyces and the corollas (Figure 2); they were uniformly arranged on the abaxial sides of both the examined floral whorls, while they were lacking on the adaxial ones (Figure 2). The small capitates are composed by a basal cell, a short unicellular stalk, and a globose or pear-shaped bicellular head (5–10 μm in diameter, Figure 2) surrounded by a thin subcuticular chamber. These hairs are generally scattered among the larger peltates on leaves, calyces, and corollas, and are the only type of trichome occurring on the leaf adaxial side, with a higher density along the midrib and lateral veins in comparison to the interveinal regions (Figure 2).

The histochemical survey on the glandular trichomes revealed the presence of lipids, terpenes, and mucilages among their secretory products (Table 2). Lipophilic substances, in particular terpenes, were exclusively observed in the cells of both the stalk and head in the peltates (Table 2). Muco-polysaccharides were solely present in the head cells of capitates (Table 2). Aluminium trichloride dye showed negative response in peltates and a strong positive reaction in small capitates (Table 2), indicating the presence of flavonoids in their secretory products.

### 3.2. Bee Records

#### 3.2.1. Bee Records at the Urban Area

On *S. yangii*, nine genera of Apoidea (*Anthidium*, *Apis mellifera*, *Bombus*, *Coelioxys*, *Halictus*, *Hylaeus*, *Lasioglossum*, *Megachile*, and *Stelis*) were detected, with only the first three common to all sites. In CL, just three genera were observed, while in PU, all nine genera were detected (Table 3). *Anthidium* was observed during 320 out of 379 patch records, outperforming the other genera in terms of frequency; at CL, with the record of a total of 3726 visits. The mean number of visits during a 5 min/patch at each site significantly changed according to sites and three genera considered (F _6,593_ = 9.94; *p* < 0.001) (Table 3). *Anthidium* was also the most detected genus, which showed an average of 15.21 ± 10.55 SD individuals observed, except in PU, where its presence was not significantly different from the other two genera considered. It was followed by *Apis mellifera* (4.04 ± 3.29 SD) and *Bombus* (2.34 ± 1.65 SD). The same results of significant differences considering both genera and sites were obtained by analysing the RA (%) of each genus (considering all genera detected) in each slot (F _27,3850_ = 20.25; *p* < 0.001). Across most surveyed sites, *Anthidium* emerged as the dominant pollinator, reaching its highest relative abundance (RA%) at CL (83.8%) and maintaining substantial dominance at BAM (56.2%), PdV (56.7%), and PU (46.5%). In contrast, *Apis mellifera* ranked second in abundance, with site-specific RA values of 31.2% (BAM), 9.5% (CL), 31.6% (PdV), and 33.8% (PU). Different values of partner diversity were found across urban sites, from a lower value in CL (0.48), BAM (0.65), and PdV (0.81) to a higher value in PU (0.98). The number of different pollinator genera followed the same trend, with lower values in CL (3), BAM (5), and PdV (7) and the highest value in PU (9).

**Figure 3:**
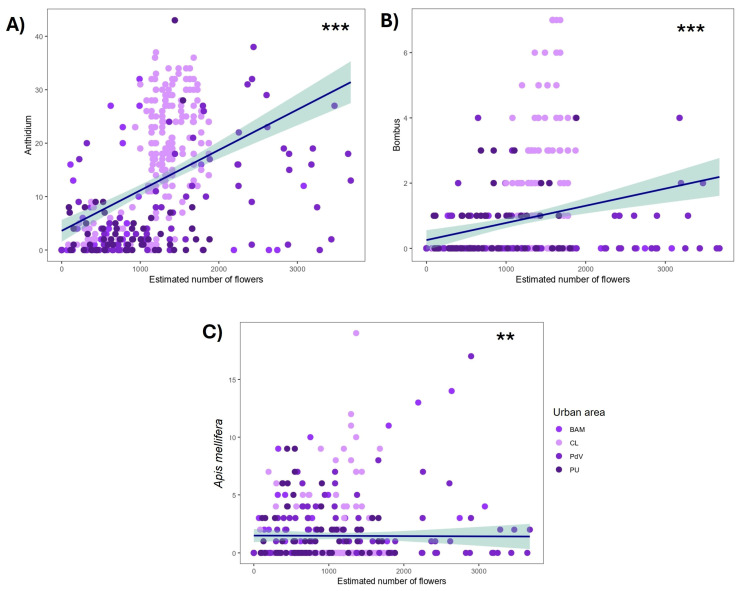
Correlations between the number of *Anthidium* spp. (**A**), *Bombus* spp. (**B**), and *Apis mellifera* (**C**) and the estimated number of flowers during each patch of observation at the four urban sites. ‘***’ *p* ≤ 0.001; ‘**’ 0.001 < *p* ≤ 0.01.

The estimated number of flowers was used as a proxy for visual floral display, which may influence pollinator attraction, and plotted against the number of Apoidea individuals (Figure 3). A clear positive trend was found in the number of *Anthidium* spp. in response to increased floral resources (ρ = 0.55; *p* < 0.001), and between the number of *Bombus* spp. and the estimated number of flowers (ρ = 0.29; *p* < 0.001), while a negative correlation was found between *Apis mellifera* and the estimated number of flowers (ρ = −0.16; *p* = 0.002).

Focal observations showed that males and females frequently moved across *S. yangii* stems in a very similar way. They either moved to a stem to rest or quickly flew among numerous stems when actively visiting flowers, largely for nectar (84 out of 99 records). Females visited on average 7.51 ± 4.41 and males 7.57 ± 3.74 stems per minute (average ± SD), with the difference not being statistically significant (t = 0.125; *p* = 0.901). Notwithstanding stems had multiple flowers in anthesis, the ratio of flowers visited per stem was mainly 1:1, with a few cases (n = 14) of 2:1 and a single case of 3:1. Males and females again behaved similarly with respect to flower visits: 6.23 ± 4.06 and 6.36 ± 3.26 visited flowers per minute (average ± SD), with no significant difference (t = 0.183; *p* = 0.855). The importance of the resource is also underlined by the resting activity of males, often recorded in a “sleeping mood” while hanging on *S. yangii* flowering stems.

#### 3.2.2. Bee Records at the Rural Area

**Figure 4:**
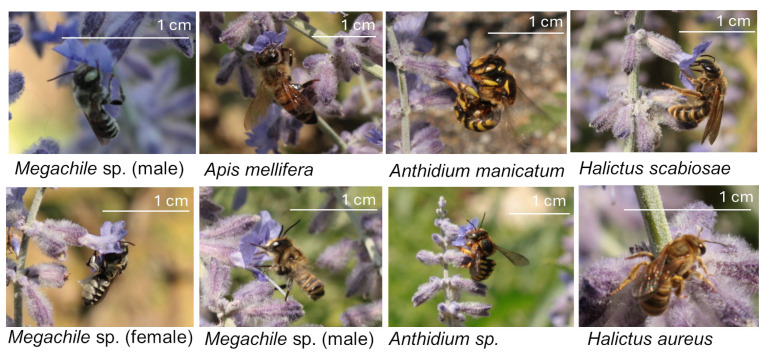
Apoidea on *Salvia yangii* at the rural site. Photo credits: M. Giovanetti.

We recorded eight genera: *Andrena*, *Anthidium*, *Apis*, *Bombus*, *Halictus*, *Hylaeus*, *Lasioglossum*, and *Megachile* (Figure 4). *Anthidium* was the second most abundant genus (29%), after the honey bee (36%), followed by *Megachile* (11%) and *Hylaeus* (9%). The remaining genus accounted for 11% of the specimens recorded. As in urban sites, the vast majority of records indicated that *Anthidium* was interested in collecting nectar; all other genera expressed the same interest.

The value of partner diversity obtained for the rural area was 1.56. Significant differences among the mean number of bees per patch in 5 min were found (F _1,7_ = 2.50; *p* = 0.03). The highest mean value was detected for *A. mellifera* (3.50 ± 0.55), while for *Anthidium*, the mean value was 2.38 ± 0.31.

**Figure 5:**
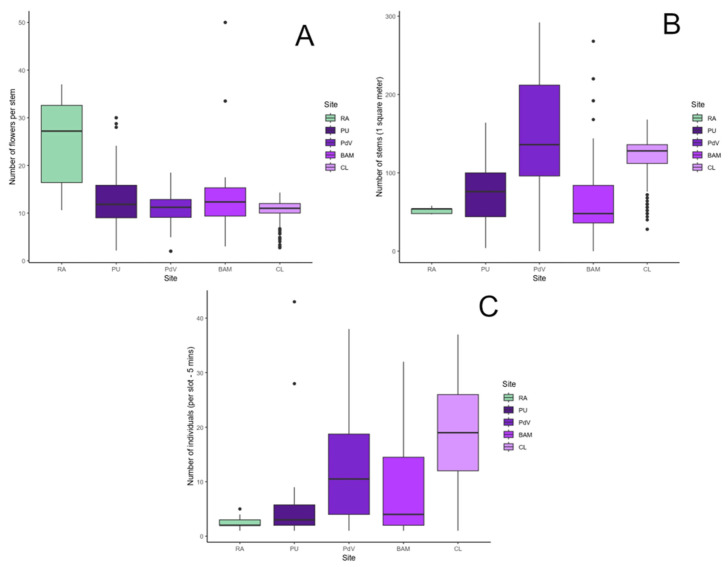
Boxplots of the number of flowers per stem (**A**); the number of stems per patch area (**B**) and the number of *Anthidium* per five-minute slot (**C**). Abbreviations refer to the following sites: the rural area (RA), Piazza Leonardo da Vinci (PdV), Parco City Life (CL), Biblioteca degli Alberi (BAM), and Piazzale Udine (PU).

At the rural site, a Kruskal–Wallis test revealed a significant difference in the number of flowers per stem among areas (H = 37.30; *p* < 0.001; df = 4; n = 333). Pairwise comparisons showed that the rural site had a significantly higher median number of flowers per stem than all other urban sites (Figure 5A). In contrast, when assessing the number of stems per plant, no significant differences were found between PU and BAM (*p* = 0.366 and *p* = 0.717, respectively), whereas both sites differed significantly from CL and PdV (*p* < 0.001 for both; Figure 5B). For *Anthidium* abundance per slot per 5 min, the Kruskal–Wallis test also indicated significant differences among sites, with pairwise comparisons showing that the rural site differed significantly from all urban sites (*p* < 0.05), except for PU (*p* = 0.347; Figure 5C).

## 4. Discussion

*Salvia yangii* appears to be a valuable resource for solitary bees of the genus *Anthidium*, as well as for other bee *taxa*, as evidenced by the observations in both urban and rural settings. Its floral morphology, particularly the approximately 5 mm corolla tube, enables efficient nectar extraction while promoting contact with reproductive structures, thereby facilitating pollination. Many generalist bee species show a preference for flowers with similar dimensions [25,26]. The attraction of various bee species to the nectar of this ornamental plant suggests its potential role in enhancing food availability in urban landscapes. The frequent occurrence of *Anthidium* spp. may be linked to the widespread planting of *S. yangii* and to the plant availability in trichomes. This is due to the fact that *Anthidium* spp., commonly known as wool-carder bees, are characterised by their unique nesting behaviour, which involves collecting plant trichomes as nesting material —particularly woolly, non-glandular hairs [7,27]. This behaviour is restricted to a few genera within the tribe Anthidiini and is associated with morphological specialisations, such as dentate mandibles for harvesting trichomes, and a dense *tomentum* on the outer basitarsi, which likely aids in absorbing secretions from glandular trichomes. These secretions are thought to be integrated into the fibrous nest material, potentially enhancing water resistance and providing antimicrobial properties that contribute to brood cell integrity [7,27].

Given these behavioural and morphological adaptations, we expected strong interest from *Anthidium* bees in plants like *S. yangii,* which exhibit a diversity of trichome types. Our findings confirmed that *S. yangii* bears both glandular and non-glandular trichomes. In particular, the woolly, non-glandular trichomes likely serve as essential nesting material, collected by females to line and partition nests within pre-existing cavities. The presence and accessibility of such plant epidermal appendages may influence habitat selection, reproductive success, and local population densities of *Anthidium* species. Anthropogenic areas with abundant trichome-rich plants could therefore support higher populations of these bees, increasing their visibility in human-dominated landscapes. However, limited information is currently available on which specific substances produced by glandular trichomes are of interest to *Anthidium* bees. Further research is necessary to elucidate the functional relationship between these secretions and bee foraging or nesting behaviour.

*Anthidium* spp. emerged as the dominant pollinators across nearly all surveyed sites, consistently outnumbering other bee genera, except at the PU location and in the rural area, where pollinator abundances were more evenly distributed. At the rural site, the markedly higher abundance of honey bees is likely attributable to nearby beekeeping activities, resulting in significantly greater numbers compared to urban sites. The consistent prevalence of *Anthidium* bees suggests that they may possess adaptive traits, such as a preference for specific floral structures or access to unique nesting materials like trichomes, that offer competitive advantages in urban environments. Notably, *Anthidium* showed a strong positive correlation with floral abundance, indicating a capacity to rapidly exploit resource-rich conditions. In contrast, *Bombus* exhibited only a weakly positive response, and *Apis mellifera* showed a slightly negative trend, potentially due to differing foraging strategies, levels of floral specialisation, or susceptibility to competition. The unique response of *Anthidium* underscores their potential role as key urban pollinators, particularly in settings where ornamental plants like *Salvia yangii* are prevalent. The analysis revealed significantly higher partner diversity in the rural site (1.56) compared to all urban sites (CL: 0.48; BAM: 0.65; PdV: 0.81; PU: 0.98), underscoring the greater complexity of interactions supported by the more heterogeneous and florally rich rural habitat. These findings indicate that, while urban green spaces can effectively sustain certain pollinator taxa, most notably *Anthidium* spp., they generally provide a narrower range of foraging opportunities. The dominance of *Anthidium* in urban environments may reflect their ability to exploit specific ornamental plants like *Salvia yangii*, but other pollinators may be limited by reduced floral diversity. From a conservation standpoint, enhancing the floral variety within urban plantings could foster richer interaction networks and support a broader spectrum of pollinator species, thereby boosting the ecological resilience and functional sustainability of urban pollination systems [28,29]. In our observations, male and female *Anthidium* spp. exhibited remarkably similar foraging behaviours on *S. yangii*. Both sexes spent comparable amounts of time per visit, typically visiting a single flower per inflorescence and a similar number of flowers within a fixed time frame. This behavioural convergence is noteworthy given the well-documented sexual dimorphism in *Anthidium* spp., where males are generally larger than females, an uncommon trait among wild bees [30]. Several hypotheses may explain this pattern. One possibility is that both males and females visit *S. yangii* flowers primarily to satisfy their own energetic needs through nectar consumption, leading to similar visitation patterns. Alternatively, the equivalence in total floral visits may arise from differing foraging strategies: males, due to their larger body size and associated higher energy demands, may visit more flowers solely for nectar intake, while females may split their visits between personal nectar intake and nectar collection for brood provisioning. These differing motivations could nonetheless yield a similar number of flower visits overall.

In many *Anthidium* species, males are active concurrently with females [31,32] and are known to engage in aggressive territorial behaviour. They defend nectar-rich floral patches by patrolling and excluding both conspecific males and heterospecific pollinators, often leveraging their larger size and, in some species, specialised abdominal spines used in combat [30]. Smaller males have been observed employing stealth tactics to approach females, attempting to evade dominant males [33]. Such chasing behaviour has been shown to disrupt plant–pollinator interactions and reduce pollinator visitation rates, both in native and introduced ranges [34]. Despite these aggressive tendencies, our findings suggest that, when foraging on *S. yangii*, male *Anthidium* behave similarly to females in terms of visitation effort, highlighting the importance of the plant as an energy resource for both sexes.

A final point to discuss may relate to the role of exotic plant species in sustaining local bee populations [35,36]. Exotic plant species can play a complex and sometimes beneficial role in sustaining local bee populations, particularly in urban and anthropogenically modified environments where native floral resources may be scarce or seasonally limited. Many exotic ornamentals provide abundant and accessible nectar and pollen, thereby extending the foraging period for generalist pollinators such as *Apis mellifera*, *Bombus* spp., and *Anthidium* spp. [12,37]. In some cases, these species fill critical gaps in flowering plant availability, supporting bee activity during periods when native plants are not in bloom [38]. However, the ecological impact of exotic species is context-dependent; while they may supplement diets and nesting resources, they can also compete with native flora and alter plant–pollinator networks [39]. When thoughtfully integrated into urban green spaces, exotic plants—particularly those with known pollinator associations—may contribute to pollinator conservation and biodiversity in fragmented landscapes. Verloove [40] documented *S. yangii* as a rare and ephemeral escape in Belgium, noting that it has been found growing spontaneously in urban environments (e.g., between paving stones and walls), particularly following hot summers. While it is not classified as invasive, its capacity to establish outside cultivated settings suggests potential for naturalisation under favourable urban conditions. Also considering the frequent xeric conditions of rural areas, it would be preferable to pay some attention to its use out of controlled garden conditions.

Ultimately, the flexibility of *Anthidium* species in exploiting exotic floral resources should be carefully considered in regions where these bees are invasive. The introduction of certain *Anthidium* species beyond their native ranges has raised ecological concerns, particularly regarding their interactions with local pollinator communities and floral resources. Several studies have investigated the potential for exploitation and interference competition in various environments, although findings remain inconclusive. For example, Soper and Beggs [41] monitored *A. manicatum* in New Zealand and documented its interactions with native flora and fauna, ultimately concluding that the species did not represent a major direct threat. Conversely, Taggar and McGrath [42] observed niche overlap between *A. manicatum* and native bee species in unmanaged urban meadows in Montreal, Canada, and reported its frequent visitation to introduced plant species, suggesting potential competition and ecological displacement. Such competition may, moreover, occur even within native ranges, where urban environments can provide ideal conditions for it to intensify. Cities often concentrate floral resources, both native and exotic, in fragmented yet resource-rich green spaces, potentially increasing encounters between *Anthidium* species and other pollinators. This may lead to heightened competition for floral and nesting resources, particularly in areas where floral diversity is low or temporal resource overlap is high.

## 5. Conclusions

This study contributed to the identification of urban habitats in Milan (Italy), such as local parks and green infrastructures, that effectively support Apoidea populations. The role of an abundant ornamental, *S. yangii,* has been investigated in detail. This plant attracted a variety of bee genera, with *Anthidium* species emerging as the most frequent visitors. Their prevalence was expected, given the resources provided by *S. yangii*: nectar accessible via a relatively short corolla tube and the presence of diverse trichomes that may serve as nesting materials. Notably, the robustness of this plant–pollinator interaction was confirmed even in a rural context, which potentially offers a wider array of floral resources and habitats. While continued monitoring is advisable, we propose that *S. yangii* may be considered for inclusion in guidelines for pollinator-friendly planting, particularly in managed or controlled urban gardening settings.

## Figures and Tables

**Table 1:** Estimates (mm) of floral parts obtained from 30 flowers of *Salvia yangii*.

	Calyx Length	Flower Length	Upper Lip Length	Lower Lip Length	Length of the Corolla Tube
Average	5.90	8.85	3.86	2.69	4.99
SD	0.51	0.50	0.24	0.15	0.46
Min	4.81	7.85	3.25	2.45	4.01
Max	6.87	9.87	4.25	2.96	5.82

**Table 2:** Results of the histochemical tests on the glandular trichomes of *Salvia yangii.* [Symbols: (−) negative response; (±) faintly positive response; (+) positive response; (++) intensely positive response.].

Stainings	Target Compounds	Capitate Trichomes	Peltate Trichomes
Fluoral Yellow-088	Total lipids	−	++
Nile Red	Neutral lipids	−	++
Nadi reagent	Terpenoids	−	++
PAS reagent	Total polysaccharides	+	−
Ruthenium Red	Acid polysaccharides	±	−
Alcian Blue	Muco-polysaccharides	+	−
FeCl_3_	Polyphenols	++	−
AlCl_3_	Flavonoids	+	−

**Table 3:** Average (±SD) number of records of each genus at the four sites, during a patch record of 5 min, with resource collected at each site and the total number of genera. Capital letters refer to the differences in the same genus among sites and lower-case letters refer to the differences at the same site among genera (*p* < 0.05). Abbreviations refer to the following urban sites: Piazza Leonardo da Vinci (PdV), Parco City Life (CL), Biblioteca degli Alberi (BAM), and Piazzale Udine (PU).

	PdV	CL	BAM	PU
*Anthidium* spp.	12.54 ± 9.68 Ba	18.91 ± 9.49 Aa	9.15 ± 9.39 BCa	5.19 ± 7.47 Ca
*Apis mellifera*	3.45 ± 2.80 Ab	5.54 ± 3.66 Ab	4.76 ± 4.11 Aab	2.89 ± 2.23 Aa
*Bombus* spp.	1.45 ± 0.94 Ab	2.96 ± 1.74 Ab	1.17 ± 0.41 Ab	1.29 ± 0.71 Aa
Resource collected	Nectar and Pollen	Nectar	Nectar	Nectar and Pollen
Number of bee genera	7	3	5	9

## Data Availability

Data can be downloaded from Lupi, Daniela; Giuliani, Claudia; Fico, Gelsomina; Malabusini, Serena; Sorvillo, Carla; Giovanetti, Manuela (2025), “Field observations and interaction data for *Salvia yangii* and *Anthidium* bees in human-modified habitats”, Mendeley Data, V1, doi: 10.17632/2ckk8p2dvh.1.

## Appendix A

Additional information on the Methods applied to trichome analyses (Section 2.2.2).

For the fresh material of plant parts (leaves, calyces, and corollas), sections ranging from 30 to 50 µm in thickness were obtained using a vibratome and/or a cryostat. Samples were also fixed in FAA solution for 48 h at 4 °C. Subsequently, fixed samples were washed in 70% ethanol for 24 h; they were then dehydrated progressively by treatment with 80% ethanol for 2 h, 95% ethanol for 2 h, and then twice in absolute ethanol for 2 h each. Pre-embedding was then performed first with ethanol and historesin in a 1:1 ratio for one night, then with a 1:2 ratio for 2 h, and in pure historesin for 3 h. Finally, the embedding was performed in a polypropylene capsule with the addition of hardener in a ratio of 1:15 of basic resin. The historesin samples were cut in 2 µm sections with an ultramicrotome. The following dyes were used [43]: Toluidine Blue as a general staining; Sudan III/IV and Fluoral Yellow-88 for total lipids; Nile Red for neutral lipids; Nadi reagent for terpenes; Periodic Acid–Schiff (PAS) reagent for total polysaccharides; Alcian Blue for mucopolysaccharides; Ruthenium Red for acidic polysaccharides; Ferric Trichloride for polyphenols; aluminium trichloride for flavonoids. Control procedures were carried out concurrently. Observations were made with a Leitz DM-RB Fluo optical microscope (Leica, Wetzlar, Germany) equipped with a digital camera (Nikon Co, Tokyo, Japan).

For each examined plant part, 10 different samples were analysed and 30 glandular trichomes and 30 internal ducts were observed. Histochemical results were assessed using a quali–quantitative scoring system that evaluates the intensity and extent of staining of the secreted materials, after being treated with specific dyes, within the subcuticular space of the glandular trichomes or the lumen of the internal ducts.

The following scoring system based on a four-point scale was used:

- Negative (−): none of the observed trichomes or ducts showed the presence of the targeted substances, which the test was designed to detect;
- Faintly positive (±): up to 10 trichomes and up to 10 ducts displayed a weak or moderate positive reaction of the secreted materials (in terms of both colour intensity and extent), implying that a low level of the targeted substances was present;
- Positive (+): 11–20 trichomes and 11–20 ducts showed the presence of the targeted substances in the form of multiple small droplets or as a single large cluster in the subcuticular spaces and within the lumen, respectively;
- Intensely positive (++): 21–30 trichomes and 21–30 ducts exhibited a very strong and clear reaction of the secreted materials, indicating the presence and high concentration of the targeted substances in the form of multiple small droplets or as a single large cluster.
